# Addressing HIV stigma in healthcare, community, and legislative settings in Central and Eastern Europe

**DOI:** 10.1186/s12981-023-00585-1

**Published:** 2023-12-11

**Authors:** Ana-Maria Schweitzer, Arian Dišković, Veronica Krongauz, Julie Newman, Janez Tomažič, Nina Yancheva

**Affiliations:** 1 Baylor Black Sea Foundation, Constanta, Romania; 2 Croatian Association for HIV and Viral Hepatitis (HUHIV), Zagreb, Croatia; 3 European Distributor Markets, Medical Department, Gilead Sciences, Uxbridge, UK; 4 HIV Strategic Implementation & Franchise, Global Medical Affairs, Gilead Sciences, Melbourne, Australia; 5 Clinic for Infectious Diseases and Febrile Illnesses, University Medical Centre Ljubljana, Ljubljana, Slovenia; 6 Department for AIDS, Specialized Hospital for Active Treatment of Infectious and Parasitic Diseases, Sofia, Bulgaria; 7 Department for Infectious Diseases, Parasitology and Tropical Medicine, Medical University of Sofia, Sofia, Bulgaria

**Keywords:** HIV, Stigma, Discrimination, CEE, Evidence-based interventions, U = U

## Abstract

**Introduction:**

Surging HIV prevalence across countries of Central and Eastern Europe (CEE) is largely a result of poor HIV care engagement and a lack of comprehensive support for key populations. This is fostered by widespread stigma across healthcare, community, and legislative settings.

**Discussion:**

Throughout CEE, HIV stigma and intersectional stigma are serious obstacles to providing adequate medical care to people living with HIV. Anticipated and enacted (experienced) stigma from healthcare professionals, and fears of breaches in confidentiality, deter individuals from having an HIV test and engaging in HIV care. Furthermore, negative connotations surrounding HIV infection can lead to discrimination from family, friends, colleagues, and the public, leading to internalized stigma and depression. Key populations that have higher HIV prevalence, such as men who have sex with men, people who inject drugs, transgender individuals, and sex workers, experience additional stigma and discrimination based on their behaviour and identities. This contributes to the concentrated HIV epidemics seen in these populations in many CEE countries. The stigma is exacerbated by punitive legislation that criminalizes HIV transmission and penalizes sexual orientation, drug use, gender identities, and sex work. Despite high levels of HIV stigma and intersectional stigma, there are many evidence-based interventions that have reduced stigma in other parts of the world. Here, we discuss the interventions that are currently being enacted in various countries of CEE, and we suggest additional effective, evidence-based interventions that will tackle stigma and lead to increased HIV care engagement and higher rates of viral suppression. We cover the promotion of the undetectable = untransmittable (U = U) message, stigma-reduction education and training for healthcare professionals, patient-centric approaches for testing and treatment, and advocacy for non-discriminatory legislation, policies, and practices. We also consider targeted stigma-reduction interventions that acknowledge the wider challenges faced by marginalized populations.

**Conclusions:**

HIV stigma and intersectional stigma in CEE drive poor engagement with HIV testing services and care. Widespread adoption of evidence-based interventions to tackle stigma highlighted in this review will improve the quality of life of people living with HIV, improve HIV care engagement, and ultimately slow the surging HIV prevalence and concentrated epidemics occurring throughout CEE.

## Introduction

Surging HIV prevalence in Central and Eastern Europe (CEE) contrasts sharply with the slowing of HIV epidemics in other regions of the world [1–3]. Across CEE, concentrated HIV epidemics are occurring among marginalized populations [4–6].

The original 90-90-90 targets of the Joint United Nations Programme on HIV/AIDS (UNAIDS) were not reached in CEE and progress to meet the updated 95-95-95 targets has been further derailed by the COVID-19 pandemic [1]. HIV stigma is commonplace within healthcare, community, and legislative settings, and is partly responsible for the missed targets [1, 3, 7–10].

Stigma presents as anticipated, enacted (experienced), and/or internalized [11]. This stigma affects engagement at every stage of the HIV care pathway, driving the high level of HIV late presenters in CEE [7] and undermining the effectiveness of HIV programmes [1]. In addition, pervasive discrimination arises from the widespread stigma and is detrimental to the physical and mental health of people living with HIV (PLWH). Further complicating the issue, intersectional stigma affects key populations of PLWH who, in addition to facing HIV stigma, also endure cultural rejection on the basis of sexual orientation, drug use, gender identities, and sex work [9].

Here, we discuss the HIV stigma and intersectional stigma experienced by PLWH in healthcare, community, and legislative settings across CEE, and the evidence-based interventions that can be used to tackle stigma and improve HIV care engagement.

## Discussion

### HIV stigma in Central and Eastern Europe

HIV stigma is problematic for HIV care engagement worldwide but, in our experience, it is particularly prevalent in CEE. Stigma can hinder HIV healthcare professionals (HCPs) from providing quality care, prevent family and friends from supporting PLWH, and stop legislators from adequately protecting human rights.

### HIV stigma in healthcare settings

When accessing healthcare, the stigma that is anticipated and experienced by PLWH is associated with increased internalized stigma [12], reduced trust in HCPs, and poor HIV care engagement [10, 13, 14].

Case reports highlight the discrimination PLWH face in CEE in the healthcare setting; for example, a patient living with HIV in Serbia stated “the worst stigma we face is from healthcare workers” [6]. PLWH in CEE are sometimes refused routine treatment at medical centres and dentists and, in some instances, endure long waiting times in isolation rooms that are subsequently disinfected. The action of refusing to treat a patient can be made on the basis that the centre or HCPs are ill-equipped to treat PLWH.

Anticipated HIV stigma from HCPs deters people from using HIV testing services and results in late presentation [10, 15]. A meta-analysis involving 10 studies from low- and middle-income countries found that individuals with high levels of perceived HIV stigma were more than twice as likely to present late for HIV care than those who perceived low stigma [16]. There is evidence of HCPs in many countries disclosing people’s HIV status without consent [5]. Consequently, fears of breaches in confidentiality lead to PLWH not disclosing their HIV status to their primary care providers [17, 18]. A study that involved interviewing 79 PLWH in Albania found that 97.4% reported fear of disclosure of their HIV status as a barrier to accessing HIV care [19].

Stigma can also arise in healthcare settings during transitional periods, such as when mothers access parent-to-child HIV transmission services [20] or when adolescent PLWH progress into adulthood [21]. Since the start of the COVID-19 pandemic, some PLWH have reported increased internalized stigma and anticipated aggravated stigma from having both HIV and testing positive for COVID-19 [22]. In some CEE countries, the pandemic has led to shortages of certain antiretroviral medications and a reduction in HIV testing [1, 23].

### HIV stigma in community settings

Discriminatory attitudes towards PLWH are widespread among the public in CEE. More than half of 15–49 year olds in Albania self-reported discriminatory attitudes towards PLWH [1], which is likely representative of attitudes in neighbouring countries. HIV stigma in the community remains a barrier to HIV care engagement, particularly for women [14, 24, 25]. Stigma also deters PLWH from disclosing their HIV status [26], which can have implications for onward transmission of HIV, treatment adherence, and an increased sense of isolation.

Anticipated and enacted HIV stigma from friends, family, and the community can lead to internalized stigma, which contributes to poor psychological health [12, 27, 28] and poor HIV care engagement. In a Ukrainian study of 204 PLWH aged 13–25 years, HIV stigma and non-disclosure of HIV status at home were associated with moderate/severe anxiety symptoms [29]. Many PLWH do not disclose their HIV status to their friends and family in anticipation of stigma, leaving them fearful of being observed taking pills and without a support system [20].

### HIV stigma in legislative settings

Punitive laws in CEE regarding HIV are driven by stigma and these laws can reduce HIV care engagement [1, 7]. Nearly all CEE countries criminalize HIV transmission or can prosecute individuals who transmit HIV using other established laws [7, 30–35]; in some countries, this extends to HIV exposure without transmission [30–32, 35]. In addition, young people’s ability to access HIV services is limited by parental consent laws in many CEE countries [36].

Laws preventing the travel of migrants with HIV can increase HIV burden and risk among migrants and within the region in which they travel [37]. Despite this, Ukrainian refugees have been granted free access to healthcare, including antiretroviral therapy, in many CEE countries [38].

### Intersectional stigma in Central and Eastern Europe

The increasing prevalence of HIV in CEE is driven by concentrated epidemics in marginalized populations, such as men who have sex with men (MSM), people who inject drugs (PWID), transgender individuals, and sex workers [4–6]. PLWH from these populations not only encounter stigma based on their HIV status but also around their identities and behaviours [4, 6, 10]. This stigma can deter many PLWHs from accessing HIV care, adopting a social support network, and maintaining a positive self-image [7, 8, 10]. Furthermore, an accurate overview of HIV in marginalized populations in CEE is challenging because of missing national data, likely a result of institutionalized discrimination and an unfavourable legal environment [8, 39–41].

Marginalized populations often report hostile or judgemental attitudes when attempting to access healthcare services [8, 10]. A study in Hungary identified the complete lack of public health programmes targeting MSM as a barrier to MSM accessing HIV services [42]. In Russia, interviews with HIV-positive PWID revealed anticipated and enacted stigma as strong deterrents to engaging with HIV services [43], and the situation is similar in CEE.

Public and political support for the improvement of HIV services for marginalized populations are limited by stigma and religious rejection of LGBTQ + identities, drug use, and sex work [1]. In addition, internalized stigma regarding homosexual sex can increase HIV-risk behaviours among MSM [44, 45]. Often forgotten, elderly PLWH also face age-related stigma that can contribute to a lack of support from family and friends [46].

Intersectional stigma is exacerbated by punitive legal environments [9]. After sub-Saharan Africa, Eastern Europe has the highest HIV prevalence (10.9%) among female sex workers globally [39]. Despite this, sex work is criminalized or subject to punitive regulation in many CEE countries [5, 47]. Reduced engagement in HIV care has been associated with legislation negatively targeting MSM [48], PWID [49], and sex workers [50].

### Interventions and initiatives tackling HIV stigma

New UNAIDS targets aim for fewer than 10% of PLWH to experience stigma and discrimination by 2025 [7]. This can only be achieved by the proactive use of stigma-reducing interventions.

Despite a lack of published studies in CEE that assess stigma-reducing interventions [51], there are examples worldwide of people-centred approaches that tackle HIV stigma leading to increased HIV care engagement [1]. Many of these approaches (Table 1) can be applied within the healthcare, community, and legislative settings of CEE. Some effective stigma-reducing interventions are already being applied within CEE, but to ensure their widespread implementation, additional resources and funding will be required. Interventions should be far-reaching and should include hospitals, doctors’ surgeries, dentists, nursing homes, needle exchange programmes, workplaces, schools, universities, religious groups, youth centres, local councils, and the family home.

**Table 1:** Examples of interventions to tackle HIV stigma in healthcare, community, and legislative settings

Stigma source	Interventions
Healthcare settings	
HCPs	• Stigma-reduction education and training • Soft-skills training • Training on the delivery of an evidence-based U = U message • Best-practice sharing, including sharing with universities and student associations of medical schools • Patient-centred approaches to care delivery, such as telehealth and self-testing at home • Routine testing for the public to reduce stigma associated with getting an HIV test • Leaflets and posters in clinics • Identifying effective interventions using the HIV Stigma Scale or other appropriate assessments • Involvement of marginalized communities in the development of educational materials and anti-stigma training • Reduction in rural/urban differences in stigma-reduction education and training
Community settings	
Public	• National and local education campaigns, in collaboration with NGOs, via marketing and media, with clear and concise communications • Promotion of the U = U message via the media and mobile phones, and at events and speeches • Stories and education shared by celebrities, artists, and key influencers • Anti-stigma campaigns via social media • Education campaigns to reduce the opinion that HIV only affects MSM or PWID • Reduction in rural/urban differences by dispelling misconceptions about HIV in rural communities through education campaigns
Employers	• Education and legal protection training
Police	• Education and training days
Parents	• Education through marketing and media campaigns • Education from schools
Schools	• Education on HIV in the context of sexual health as part of the school curriculum, delivered by members of NGOs, trained HCPs, or well-trained teachers if appropriate • HIV education training programmes for teachers • Promotion of non-discriminatory use of language in educational materials
Religious groups	• Engagement with religious leaders to improve education and inclusivity for PLWH
Internalized stigma	• Community-based apps providing a safe and private platform to share stories • Leaflets from HCPs
Legislative settings	
Laws	• Advocation against discriminatory or unfavourable legislation, policies, and practices that target PLWH or marginalized communities • Campaigns for the decriminalization of HIV transmission and sex work • Promotion of non-discriminatory use of language in legislation
Legal aid	• Legal counselling services to address a lack of legal aid for PLWH and for marginalized communities
Politicians and policymakers	• Advocation for state support in anti-stigma programmes and services • Engagement with political leaders to improve inclusivity of PLWH in legislation and budgets • Campaigns for policy change within institutions, such as universities and workplaces

HCP: healthcare professional; MSM: men who have sex with men; NGO: non-government organization; PLWH: people living with HIV; PWID: people who inject drugs; U = U, undetectable = untransmittable

The promotion of the undetectable = untransmittable (U = U) message is key to tackling HIV stigma in local communities in CEE, and it needs to be disseminated through multiple modalities. Stigma has been impeding the communication of the U = U message from HCPs to patients and its acceptance by the public [52, 53], but an evidence-based U = U message can lower stigmatized attitudes [54]. U = U awareness will also likely reduce anticipated HIV stigma relating to dating and sex [55], improving the quality of life (QoL) of PLWH. For example, among patients enrolled in long-term multidisciplinary services at the Baylor Black Sea Foundation in Romania, > 70% of PLWH were aware of U = U and > 70% reported a good QoL [56]. U = U messaging should be promoted in all HIV care guidelines within CEE. In addition, training on patient–provider communication of U = U and the evidence supporting U = U should form part of the education of all HIV care providers.

Monitoring the effectiveness of HIV stigma-reducing interventions using validated measures can support local programmes advocating for additional resources and enable the identification and sharing of best practice. The HIV Stigma Scale is a reliable and validated measure of HIV-related stigma [57], which could be adapted for use in CEE in the same way that it has been in Turkey [58, 59], Ukraine [29], and Japan [60]. An adapted HIV Stigma Scale to monitor stigma related to pre-exposure prophylaxis use has also been developed [61]. In healthcare settings, the Health Care Provider HIV/AIDS Stigma Scale can be used to prioritize training needs, draft interventions, and assist HCPs with self-assessing their stereotyping, prejudiced beliefs, and discriminatory behaviours [62]. Periodic assessments of stigma levels experienced by patients can be used to develop strategic quality improvement objectives.

### Interventions and initiatives in healthcare and settings

Highly effective stigma-reducing interventions can be implemented across CEE to address stigma in healthcare and community settings. Best-practice sharing and soft-skills training can reduce enacted stigma from HCPs. In particular, interventions focused on training popular opinion leaders among HCPs have been effective in reducing HIV stigma and discrimination [63].

It is essential that HCPs have an accurate understanding of HIV transmission and prevention. The Bulgarian Scientific Society of Infectious Diseases organizes training for HCPs on these topics with the aim of reducing associated stigma towards patients. Likewise, the Croatian Association for HIV and Viral Hepatitis reduces HIV stigma among HCPs via organized training on the needs of PLWH and the challenges and legal issues they face. HCPs are also encouraged to become involved with their initiatives and campaigns. Simple educational materials can also be used to empower patients. For example, a local-language leaflet distributed to PLWH in Slovenia included advice on their legal rights regarding personal information protection, privacy, and treatment, and also provided instructions on how to report HIV stigma and/or discrimination [64].

Patient-centric healthcare approaches, such as telehealth and self-testing at home, which have been used to reduce the risk of COVID-19 transmission and promote privacy, can be utilized for HIV care. These approaches will likely reduce avoidance of HIV services because of anticipated HIV stigma in healthcare settings [65].

Fostering relationships between organizations running anti-stigma initiatives and the media can lead to campaigns that disseminate anti-stigma messages to a wide audience. These campaigns can address the HIV stigma ingrained in communities, which largely originates from media coverage of HIV in the 1980s. Media campaigns can involve celebrities/influencers and utilize social media, television, and newspapers. The average person in CEE spends over 3.5 h daily watching television [66], and 68–82% of people in CEE have smartphones [67]. For young PLWH, apps can provide a safe virtual space to engage with other PLWH, helping to reduce internalized stigma [68]. Young PLWH using a community-based app in the USA were able to open up about intense internalized stigma, such as bathing in bleach to ‘feel more clean’ and like they ‘don’t have HIV’ [68].

In the workplace, HIV stigma-reduction education and training for employers can be used to reduce stigma that is anticipated and experienced by PLWH when seeking or maintaining employment [69].

### Interventions and initiatives in legislative settings

To tackle punitive laws that entrench discrimination, interventions need to reduce stigma among legislators and stimulate public support. Punitive legislation needs to be adapted so that it empowers PLWH to defend their human rights, health, and safety. Advocation for legislation and policy change is most effective with the involvement of patients who can share real-life stories.

Change can be advocated for through the media and by working with governments and non-government organizations (NGOs). Examples include the formation of national coalitions against HIV criminalization, the promotion of legal aid for PLWH, and the monitoring of human rights violations. NGOs in Slovenia successfully advocated for penalties for dentists who discriminate against their patients with HIV. In Romania, the Baylor Black Sea Foundation, alongside social workers and lawyers, empowers PLWH to use and advocate for their legal rights [64].

### Interventions and initiatives tackling intersectional stigma

There is an urgent need for more effective intersectional stigma interventions in CEE. In healthcare and community settings, interventions to tackle intersectional stigma must address the wider challenges faced by marginalized populations. HCPs who offer unbiased care to PLWH from marginalized communities will build strong relationships with their patients, which can improve HIV care engagement.

Examples of interventions that address intersectional stigma in the healthcare setting include stigma-reduction and soft-skills training and unbiased U = U message communication training. In addition, enacted intersectional stigma can be challenged in the healthcare setting by repercussions for staff who display stigma-related discrimination towards patients.

Targeted interventions can be effective in healthcare and community settings. For instance, Teenergizer, a youth organization supporting teenagers with HIV across Eastern Europe, helps young people to navigate life with HIV and to deal with the associated stigma and discrimination [4]. In Russia, a community-based, adapted form of acceptance and commitment therapy aims to tackle internalized stigma experienced by HIV-positive PWID by providing a space outside formal healthcare settings [70]. This approach could also be used in CEE.

NGOs that work with marginalized communities should include anti-stigma and psychological support as part of their services. For instance, CheckPoint, an NGO operating in Bulgaria that works with MSM, offers psychosocial support alongside traditional HIV testing services and treatment. The interdisciplinary cooperation of specialized NGOs, HCPs, and charities, through interventions like field visits, community testing, education, and conferences, can be highly effective in combating stigma and discrimination. Additionally, the inclusion of marginalized communities in the development and execution of stigma-reducing interventions can improve their overall effectiveness [1].

To tackle intersectional stigma among the public, marketing campaigns, including television and community-based apps, can be used to promote acceptance and to advocate for changes in punitive legislation affecting marginalized communities. Modelling predicts that 33–46% of new HIV infections among sex workers and clients over a decade could be prevented by decriminalizing sex work [71].

Interventions tackling intersectional stigma can be monitored by using validated measures with expanded scopes, such as the People Living with HIV Stigma Index 2.0 [72, 73] and the Experiences of Sex Work Stigma scale [74]. These tools may help to identify successful stigma-reducing interventions that are already improving the lives of PLWH from marginalized communities, so that best practice can be shared across CEE.

## Conclusions

HIV stigma in CEE drives poor engagement with HIV care and contributes to inconsistent antiretroviral therapy adherence, which ultimately results in low viral suppression and poor health outcomes for PLWH. Marginalized populations in CEE – such as MSM, PWID, transgender individuals, and sex workers – have a higher HIV burden but poorer access to HIV care because of intersectional stigma. Evidence-based interventions are being used to tackle HIV stigma and intersectional stigma. Further widespread adoption of these interventions will improve the QoL of PLWH, improve HIV care engagement, and ultimately slow the increasing HIV prevalence and concentrated epidemics occurring throughout CEE. Future anti-stigma interventions need to be well designed, with detailed descriptions of their content to allow for replication and critical evaluation [75].

## Data Availability

Not applicable.

## Abbreviations

AIDS: acquired immunodeficiency syndrome
CEE: Central and Eastern Europe
COVID-19: coronavirus disease 2019
HCP: healthcare professional
HIV: human immunodeficiency virus
LGBTQ+: lesbian, gay, bisexual, transgender, queer, plus
MSM: men who have sex with men
NGO: non-government organization
PLWH: people living with HIV
PWID: people who inject drugs
QoL: quality of life
UNAIDS: the Joint United Nations Programme on HIV/AIDS
U = U: undetectable = untransmittable
